# A Systematic Review of Flurbiprofen 8.75 mg Dose and Risk of Haemorrhagic Events

**DOI:** 10.3389/fphar.2021.726141

**Published:** 2021-08-04

**Authors:** Sandeep Dhanda, Alison Evans, Debabrata Roy, Vicki Osborne, Adam Townsley, Graça Coutinho, Anuradha Kulasekaran, Saad Shakir

**Affiliations:** ^1^ Drug Safety Research Unit, Southampton, United Kingdom; ^2^ University of Portsmouth, Portsmouth, United Kingdom; ^3^ Global Pharmacovigilance, Reckitt Benckiser Health Limited, Slough, United Kingdom; ^4^ Global Medical Affairs, Reckitt Benckiser Health Limited, Slough, United Kingdom

**Keywords:** flurbiprofen, lozenge, oromucosal spray, haemorrhage, bleeding

## Abstract

Oral non-steroidal anti-inflammatory drugs (NSAIDs) are known to be associated with an increased risk of bleeding. The NSAID, flurbiprofen, in the form of 8.75 mg lozenge or oromucosal spray is indicated for the symptomatic relief of sore throat. Despite the low dose as compared to alternative flurbiprofen preparations, concerns have been raised regarding its safety in terms of haemorrhagic events. This systematic review was conducted to identify existing evidence on the risk of haemorrhagic events with flurbiprofen 8.75 mg dose (any formulation), particularly where this may be due to potential interactions with other medicinal products. The systematic review examined studies reporting haemorrhagic events in patients receiving flurbiprofen 8.75 mg dose. Six individual electronic databases were searched up to 28th April 2020. Records were initially screened for relevance followed by further review of potentially eligible studies. Data extraction was performed for eligible studies and risk of bias in studies was assessed. The search strategy identified 1093 individual records. Of these, 1038 records were excluded after initial review; the majority of these records related to flurbiprofen in alternative formulations with alternative doses (e.g., eye drops, skin patches, oral tablets) thus were not considered relevant for further review. The 55 remaining records related to flurbiprofen 8.75 mg dose (any formulation) or flurbiprofen lozenge/oromucosal spray where the dose was not specified. After further review, 52 of these records were not considered eligible. Thus, only three records were included in this systematic review. The three studies reported a total of five haemorrhagic events in patients taking flurbiprofen 8.75 mg lozenge; the corresponding risk in each of the studies was 8.33, 1.98 and 1.96%. Where possible, comparison of flurbiprofen 8.75 mg lozenge to placebo produced risk ratios of 0.96 (95% CI 0.07, 13.25) and 2.00 (95% CI 0.10, 118.0). This systematic review found limited evidence on the risk of haemorrhagic events with flurbiprofen when used at a dose of 8.75 mg. Counts were low across all studies and results comparing flurbiprofen and placebo treatment arms were non-significant. However, scarcity of studies and low certainty of evidence for the outcome of haemorrhagic events limits the conclusions of this systematic review.

## Introduction

Flurbiprofen, a NSAID, is a mixed cyclo-oxygenase (COX)-1/COX-2 inhibitor with some selectivity towards COX-1, thereby inhibiting the synthesis of prostaglandins which play a key role in the generation of the inflammatory response (Ricciotti and FitzGerald, 2011). It is licensed, in a tablet form, to treat a range of musculoskeletal conditions including rheumatoid arthritis and osteoarthritis. Furthermore, its analgesic effect has proven efficacy for the relief of mild to moderate pain, related to conditions such as dental pain and post-operative pain (Reckitt Benckiser Healthcare (UK) Ltd, 2016). It is the combined local and systemic effects achieved via local delivery of flurbiprofen to the inflamed tissues of the pharynx which has resulted in the development of flurbiprofen 8.75 mg in a number of different formulations (including lozenge and spray) for sore throat treatment (Reckitt Benckiser Healthcare (UK) Ltd, 2016).

Sore throat is one of the most common reasons for primary healthcare consultations (Gulliford et al., 2014). The majority of cases have a viral aetiology which are most often self-limiting, however for ease of symptoms several self-care management approaches have been recommended by The National Institute for Health and Care Excellence (NICE) 2018 including medicated over the counter (OTC) products that can provide relief (National Institute for Health and Care Excellence, 2018). Flurbiprofen lozenge, marketed since 1999 and first licensed in the European Union (EU) in 2006, is indicated for the short term symptomatic relief of sore throat in adults and children over the age of 12 years; the oromucosal spray is intended for use in adults aged 18 years and over excluding Russia with paediatric use from age 12 years and above (Reckitt Benckiser Healthcare (UK) Ltd, 2016; Reckitt Benckiser Healthcare (UK) Ltd, 2018).

A single dose of either formulation relieves sore throat, through a significant reduction in sore throat pain intensity, reduction in difficulty swallowing and feeling of a swollen throat (Reckitt Benckiser Healthcare (UK) Ltd, 2018; Reckitt Benckiser Healthcare (UK) Ltd, 2016).

Despite its proven benefits, flurbiprofen is susceptible to adverse events commonly associated with the COX enzyme inhibition properties of NSAIDs. In particular, the COX-1 enzyme is involved in gastroprotection from gastric acid and in thromboxane formation which stimulates platelet aggregation and blood clot formation (Rao and Knaus, 2008; Moore et al., 2015). Thus, inhibition of this enzyme can increase the risk of bleeding, in particular gastrointestinal (GI) bleeding. In many cases these adverse events may occur because of drug-drug interactions (DDIs) between flurbiprofen and other medicinal products such as other NSAIDs, anticoagulants or corticosteroids (Reckitt Benckiser Healthcare (UK) Ltd, 2016). Therefore, consideration of the use of concomitant medications is important when assessing the potential risk of haemorrhagic events with flurbiprofen.

The aim of this study was to collate all existing evidence on haemorrhagic events occurring in patients taking flurbiprofen 8.75 mg dose (any formulation). The primary objective was to identify the frequency of haemorrhagic events occurring with flurbiprofen 8.75 mg dose (any formulation). Secondary objectives included describing the severity of the haemorrhagic events, comparing occurrence with comparator arms (e.g. other NSAIDs) and investigating whether haemorrhagic events on flurbiprofen 8.75 mg occur as a consequence of potential DDIs.

## Methods

### Inclusion Criteria for Studies

The study designs considered appropriate for inclusion were clinical trials (randomised and non-randomised, blinded and non-blinded), cohort studies (prospective and retrospective), case-control studies, cross sectional studies, case series and case reports.

The inclusion criteria followed the PICOS (Participants, Interventions, Comparators, Outcomes, and Study design) categories outlined in the Preferred Reporting Items for Systematic Reviews and Meta-Analyses (PRISMA) statement (Table 1) (Liberati et al., 2009; Page et al., 2021). No time limits were applied and all literature on flurbiprofen 8.75 mg (any formulation) since inception up to the time of the electronic search (data lock: 28th April 2020) were considered for inclusion.

**TABLE 1:** Inclusion criteria.

**Participants**
All patients (any age) taking flurbiprofen 8.75 mg dose (any formulation) in any setting (primary and/or secondary care)
The population was not restricted to the UK and included international studies
**Intervention (Exposure)**
Reported use of flurbiprofen 8.75 mg dose (any formulation) prescribed for any indication prescribed alone or in combination with other medicinal products
**Comparator**
A study comparator group was not required for inclusion. However, where a comparator group was specified, this could have been placebo or an active comparator. Active comparators could include NSAIDs:
• Of any formulation (i.e., oral, topical)
• Prescribed for any indication
• Within any of the three categories of sales (General sales list (GSL), Pharmacy only (PO), Prescription only medicines (POM))
• Prescribed alone
• Prescribed in combination with other medicinal products
The active comparator group could also include:
• Flurbiprofen 8.75 mg dose (whichever is not the specific exposure formulation) alone
• Flurbiprofen 8.75 mg dose (whichever is not the specific exposure formulation) plus other medicinal products
• Flurbiprofen 8.75 mg dose (where it is the same as the specific exposure formulation) plus other medicinal products
• Flurbiprofen at any other dose (other than 8.75 mg) alone and in any formulation
• Flurbiprofen at any other dose (other than 8.75 mg) in any formulation plus other medicinal products
**Outcomes**
Studies were included if they reported a haemorrhagic adverse event. The haemorrhagic event could have occurred:
• In any anatomical site
• At any severity
• With flurbiprofen 8.75 mg dose (any formulation) alone
• With flurbiprofen 8.75 mg dose (any formulation) plus other medicinal products
• With the comparator drug alone (where comparator group available)
• With comparator drug plus other medicinal products (where comparator group available). Studies with bleeds reported only in the comparator arm (and not in the flurbiprofen arm) were considered for inclusion.
**Study design**
The following study designs were eligible for inclusion in the systematic review:
• Clinical trials (randomised and non-randomised, blinded and non-blinded)
• Cohort studies (prospective and retrospective)
• Case-control studies
• Cross sectional studies
• Case series
• Case reports

### Exclusion Criteria for Studies

The following exclusion criteria were applied; research in languages other than English, studies only specifying adverse events but not specifically stating haemorrhagic events, pre-clinical studies, reviews (however reference lists from relevant reviews were examined to identify any other eligible papers for inclusion), conference abstracts.

### Search Strategies

A systematic electronic search of the following databases was performed; PubMed/MEDLINE, Embase, The Cochrane Library, Web of Science, ClinicalTrials.gov (http://clinicaltrials.gov.), EU Clinical Trials Register (https://www.clinicaltrialsregister.eu/ctr). No restrictions in terms of dates of coverage were applied. The search strategy was restricted to human studies only (this was possible for PubMed and Embase but not possible for Web of Science and The Cochrane Library). For Embase and Web of Science, the search was restricted to articles only.

The following search strategy was used where possible; for ClinicalTrials.gov only “Flurbiprofen” search concept was used:

#### Search Concept 1:

Flurbiprofen (including all synonyms for this concept) AND

#### Search Concept 2:

Lozenge OR oromucosal spray (including all synonyms for these concepts e.g., spray, buccal, oromucosal) OR 8.75

To ensure identification of all possible cases a further search was performed using search concept 1 but restricting to case reports only. This was only possible for PubMed.

A complete description of the search strategy used for each database (including filters applied) has been provided in the Supplementary Material: Search Strategy.

### Additional Searches

A manual search was performed for other studies using references cited from papers which were considered relevant but not found from the systematic electronic database search. The publicly available EudraVigilance database was investigated to identify potential further cases. Furthermore, the European Medicines Agency (EMA) website was reviewed for any additional case reports.

### Study Selection

Studies retrieved from the electronic search were initially de-duplicated. The remaining studies were reviewed independently by two reviewers; initial review of records (i.e., titles and abstracts, plus full text where required) resulted in a subset of papers that were further fully reviewed for eligibility. Any discrepancies between the two reviewers were discussed and where necessary adjudicated by a third reviewer. For papers considered to be ineligible, the reason for exclusion was recorded. All references were managed in EndNote, with the exception of ClinicalTrials.gov and EU Clinical Trials where it was not possible to export to EndNote.

### Data Extraction

Data was extracted from eligible studies into a data extraction form by the two reviewers independently according to the key data items outlined in the Supplementary Material: Data items for extraction. For categories where the information was not reported, this was classified as not specified. Where possible, an attempt was made to seek further information or clarification from the authors if required.

### Risk of Bias Assessment in Individual Studies

Each included study was assessed for risk of bias according to the guidelines outlined in the Cochrane Handbook (Higgins and Thomas, 2019). The aim was to use the Cochrane Risk-of-Bias (RoB2) tool and the Risk Of Bias In Non-randomised Studies - of Interventions (ROBINS-I) tool to assess risk of bias in the results of randomised trials and non-randomised studies, respectively (Sterne et al., 2019; Sterne et al., 2016). For case reports, the aim was to evaluate their methodological quality (Murad et al., 2018). However, no non-randomised trials or case reports were identified for the systematic review.

The five bias assessment domains in the RoB2 tool are the risk of bias arising from; 1) the randomisation process, 2) deviations from the intended interventions (effect of assignment to intervention), 3) missing outcome data, 4) measurement of outcome, 5) the selection of the reported result. Each domain was judged as either “low risk”, “high risk” or “some concerns” of bias. Where studies were allocated one or more “high risk” criteria or “some concerns” for multiple domains, the study was classed as having an overall high risk of bias. A study is allocated to “some concerns” if the study was judged to raise “some concerns” in at least one domain, but not to be at “high risk” for any domain.

### Certainty of Evidence

Certainty of evidence for safety (risk of haemorrhagic events) was assessed post hoc using the Grading of Recommendations, Assessment, Development and Evaluations (GRADE) framework (Balshem et al., 2011). The following GRADE domains were assessed; risk of bias, imprecision, inconsistency, indirectness and other considerations (publication bias, large effect, plausible confounding and dose response gradient). Each domain was judged, and an overall certainty of evidence calculated.

### Synthesis of Results

Results were summarised in narratives, tables and figures, with treatments and outcomes reported as specified by authors in the individual studies. For studies where only counts were specified, an attempt was made to calculate measures of frequency (e.g., risk) if the appropriate numerator and denominator information was provided.

## Results

### Search Results

**FIGURE 1:**
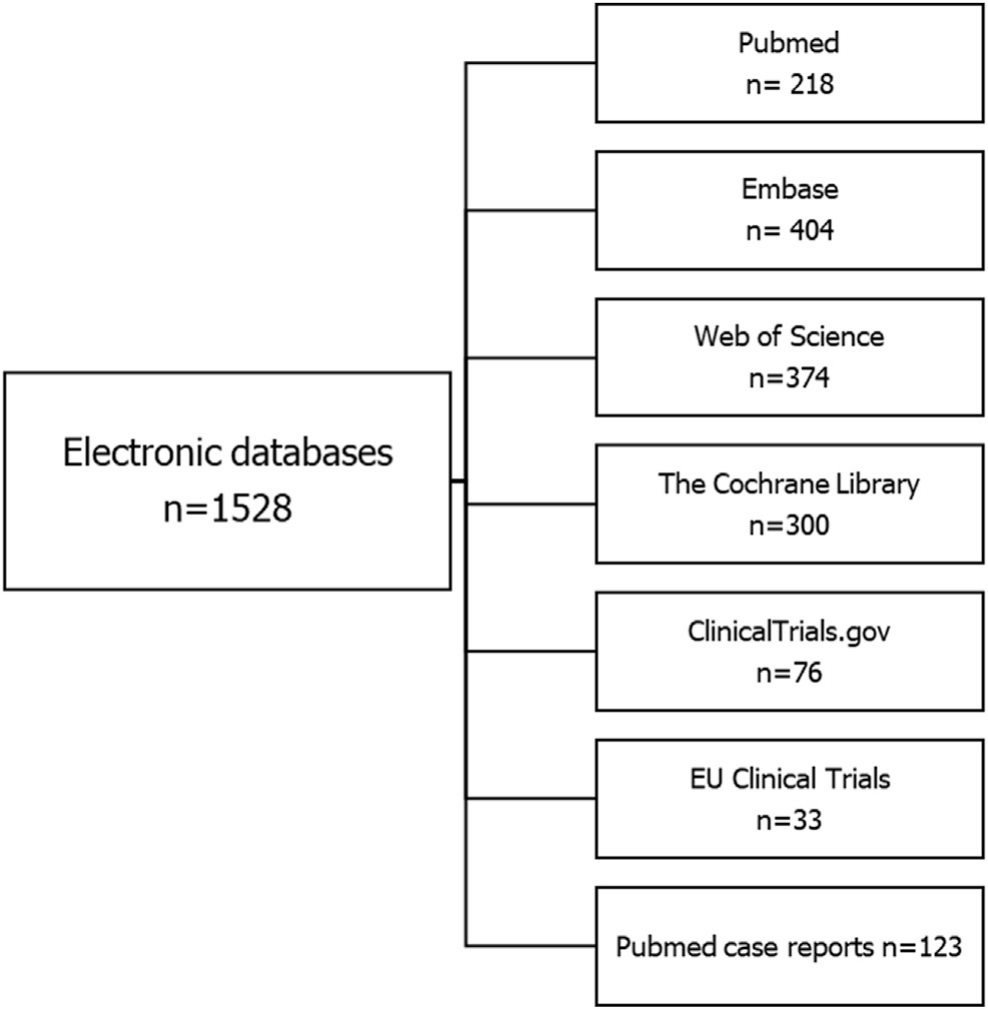
Results of electronic search (up to 28^th^ April 2020).

**FIGURE 2:**
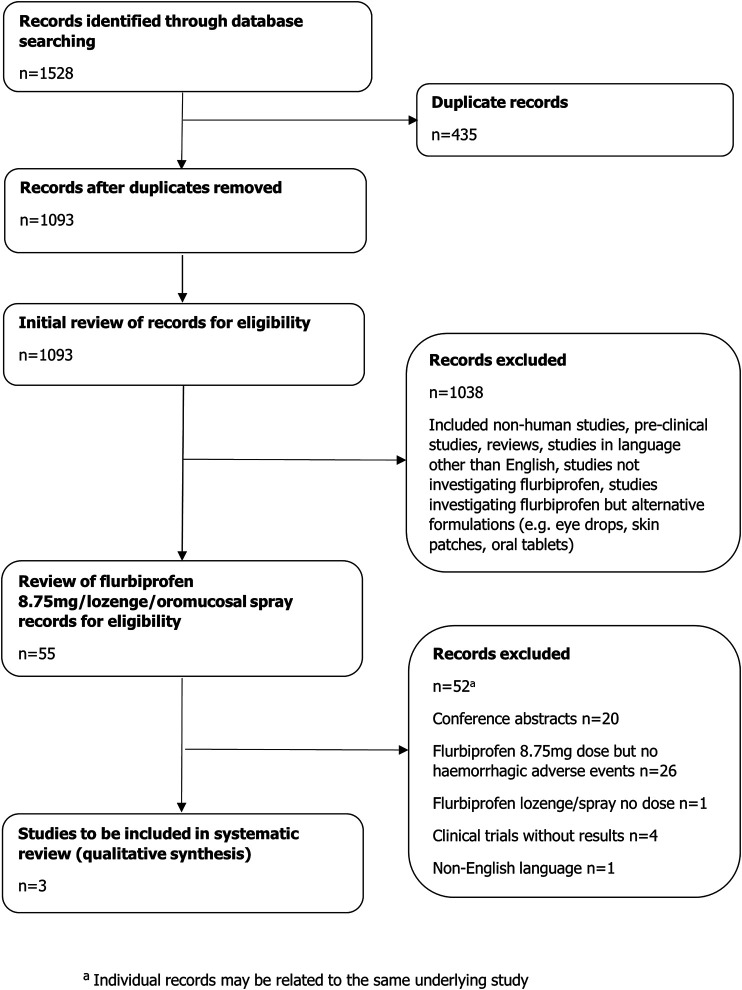
Flow diagram of study selection.

The search strategy identified 1,528 citations across the individual electronic databases (Figure 1). After 435 duplicates were removed, the authors screened the remaining 1,093 records to assess for eligibility (Figure 2).

The initial stage of screening excluded 1,038 records; the majority of these records related to flurbiprofen alternative formulations not at 8.75 mg dose (e.g., eye drops, skin patches, oral tablets) and thus were not considered relevant for further review. Other reasons for exclusion have been listed in Figure 2.

The 55 records remaining after the initial screening related to flurbiprofen 8.75 mg dose (any formulation) or flurbiprofen lozenge/oromucosal spray where the dose was not specified. Of these, 52 records were not considered eligible. Reasons for exclusion have been listed in Figure 2. Thus, only three records met the inclusion criteria for the final analysis; these were studies reporting haemorrhagic events with flurbiprofen 8.75 mg.

### Results of Eligible Studies From Electronic Database Search

The eligible studies included a cross-over trial and two double-blind placebo-controlled trials. In the randomised, two-period, cross-over, open-label study evaluating the pharmacokinetic profiles of two different oromucosal flurbiprofen 8.75 mg lozenges in 12 healthy volunteers, *Matzneller et al* reported one haemorrhagic event, specified as “haematoma”; risk 8.33% (*n* = 1) (Table 2) (Matzneller et al., 2012).

**TABLE 2:** Summary of studies reporting haemorrhagic events with flurbiprofen 8.75 mg.

Data	Study 1	Study 2	Study 3
Author	Matzneller et al.	NCT01048866	NCT01049334
Type of study	Randomised two-period cross-over open label trial	Randomised double-blind placebo-controlled trial	Randomised double-blind placebo-controlled trial
Country	Austria	United States	United States
Year	2010	2011	2011
Population	Healthy volunteers aged 18–55 years	Patients >18 years with sore throat due to acute pharyngitis	Patients >18 years with painful pharyngitis
Exposure	Flurbiprofen 8.75 mg compressed lozenges (Alfa Wasserman S.p.A)	Sugar-based, flavoured flurbiprofen 8.75 mg lozenge	Flurbiprofen 8.75 mg lozenge
Comparator	Flurbiprofen 8.75 mg lozenges (Benactiv Gola^®^, Reckitt Benckiser Healthcare)	Sugar-based, flavoured matching placebo lozenge	Placebo lozenge
Primary outcome	Bioavailability (pharmacokinetic profile)	Time weighted sum of pain intensity difference in sore throat pain intensity scale over 24 h post baseline	Time weighted sum of pain intensity difference in sore throat pain intensity scale over 24 h post baseline
Follow up duration	24 h after dose (for primary outcome)	7 days	7 days
Subjects (*n*)	Total *n* = 12	Total *n* = 198;	Total *n* = 204;
		Flurbiprofen *n* = 101	Flurbiprofen *n* = 102
		Placebo *n* = 97	Placebo *n* = 102
Age (mean) years	23 years	33.9 years	19.8 years
		Flurbiprofen 33.5 years,	Flurbiprofen 19.8 years,
		Placebo 34.2 years	Placebo 19.8 years
Sex^a^ (*n*; %)	8 f (66.7%), 4 m (33.3%)	119 f (60.1%), 79 m (39.9%)	117 f (57.4%), 87 m (42.6%)
		Flurbiprofen 61 f, 40 m	Flurbiprofen 54 f, 48 m
		Placebo 58 f, 39 m	Placebo 63 f, 39 m
Haemorrhagic event (*n*)	Total *n* = 1 Haematoma	Total n = 4;	Total *n* = 3; Flurbiprofen: epistaxis (*n* = 2), Placebo: epistaxis (*n* = 1)
		Flurbiprofen: haematochezia (*n* = 1), epistaxis (*n* = 1)	
		Placebo: mouth haemorrhage (*n* = 1), epistaxis (*n* = 1)	
Risk of haemorrhagic event (%)^b^	8.3%	Flurbiprofen 1.98%^c^	Flurbiprofen 1.96%^c^
		Placebo 2.06%^c^	Placebo 0.98%^c^
Risk ratio	n/a	0.96 (95% CI: 0.07, 13.25)^d^	2.00 (95% CI: 0.10, 118.0)^d^

a f = female, m = male.

b Overall risk estimates for each treatment arm not provided in results; calculated manually.

c An individual patient may have experienced more than one haemorrhagic event so risk has been calculated as number of haemorrhagic events/number of patients at risk.

d Risk ratio calculated manually as risk in flurbiprofen treatment arm/risk in placebo lozenge arm.

This second eligible study (NCT01048866) was a multi-centre randomised double-blind placebo-controlled trial comparing the safety and efficacy of flurbiprofen 8.75 mg lozenge to placebo lozenge in patients >18 years old with painful pharyngitis (Table 2) (ClinicalTrials.gov. National Library of Medicine (United States), 2010a). Over the 7 day treatment period, there were two cases of bleeding events in the flurbiprofen lozenge arm; these were reported as “haematochezia” (*n* = 1, 0.99%) and “epistaxis” (*n* = 1, 0.99%). In the placebo lozenge arm, there were also two bleeding events reported as “mouth haemorrhage” (*n* = 1, 1.03%) and “epistaxis” (*n* = 1, 1.03%). All events reported in this trial were classified as non-serious as reported by the authors.

In the single-centre randomised double-blind placebo-controlled trial comparing the analgesic efficacy of flurbiprofen 8.75 mg lozenge to placebo lozenge and the safety of flurbiprofen throughout the course of treatment of sore throat due to acute pharyngitis (NCT01049334), a total of three bleeding events were reported during the 7 day treatment period (Table 2) (ClinicalTrials.gov. National Library of Medicine (United States), 2010b). All were reported as “epistaxis” and classified as non-serious by the authors; two cases occurred in the flurbiprofen lozenge arm (1.96%) and one (0.98%) in the placebo lozenge arm. Further information on each study can be found in the Supplementary Material: Results of eligible studies from electronic database search.

### Results of Additional Searches

A manual search for other studies using any references cited from papers which were considered relevant revealed no additional studies to those already found from the electronic database search.

Review of EudraVigilance was limited by a lack of differentiation between flurbiprofen dosages and formulations (i.e., tablet, lozenge, oromucosal spray), thus it was not possible to use these data sources to identify further cases. The EMA website was reviewed for any additional case reports, however, none were found.

### Risk of Bias in Eligible Studies From Electronic Database Search

The three studies eligible for inclusion in the systematic review were assessed for risk of bias. Results have been summarised in Table 3. Overall, all three studies were judged as having “some concerns” regarding risk of bias.

**TABLE 3:** RoB2 assessment for included studies.

Domain	Description	Study 1	Study 2	Study 3
		*Matzneller et al*	*NCT01048866*	*NCT01049334*
1	Risk of bias arising from the randomisation process	Low risk	Low risk	Low risk
2	Risk of bias due to deviations from the intended interventions (effect of assignment to intervention)	Low risk	Low risk	Low risk
3	Missing outcome data	Low risk	Low risk	Low risk
4	Risk of bias in measurement of outcome	Low risk	Low risk	Low risk
5	Risk of bias in the selection of the reported result	Some concerns	Some concerns	Some concerns
Overall risk of bias		Some concerns	Some concerns	Some concerns

### Results of Certainty of Evidence of Eligible Studies from Electronic Database Search

The certainty of evidence for the safety outcome of haemorrhagic events was assessed as “very low”. Results are presented in Supplementary Material: GRADE Evidence Profile table.

### Results of Studies Identified from Electronic Database Search Reporting Flurbiprofen at Other Low Doses

There were additional studies (*n* = 3) retrieved from the electronic search which reported haemorrhagic events with flurbiprofen (in formulations synonymous with oromucosal spray/lozenge) but at a low dose other than 8.75 mg (Türk et al., 2018; Isler et al., 2018; Dionne et al., 2004). These did not fulfil the eligibility criteria for the systematic review. All three studies were randomised controlled trials evaluating alternative low doses of flurbiprofen use (other than the 8.75 mg dose or where dose was not specified) in patients undergoing oral surgery. Bleeding events were described as post-operative haemorrhage or delayed bleeding.

## Discussion

### Summary of Main Findings

This systematic review examined current evidence on the risk of haemorrhagic events with flurbiprofen 8.75 mg dose, in all formulations. The primary objective of the systematic review was to identify the frequency of haemorrhagic events occurring with flurbiprofen 8.75 mg dose. Secondary objectives included describing the severity of the haemorrhagic event(s) and comparing the frequency of haemorrhagic events with flurbiprofen 8.75 mg to the frequency occurring with a comparator group, where available.

In total, 1,093 individual records were identified across six electronic databases, of which 1,038 records reported flurbiprofen at alternative formulations (e.g., eye drops, oral tablets) which were of different dosages to 8.75 mg (the majority being higher). The remaining 55 records included flurbiprofen 8.75 mg (any formulation) or flurbiprofen lozenge/oromucosal spray where the dose not was specified. Of these, 52 records were excluded, most of which did not report a haemorrhagic adverse event in the paper or were conference abstracts. Thus, from our search, we identified only three records reporting haemorrhagic events considered eligible for inclusion in this systematic review.

One haemorrhagic event (8.33%) was reported in a randomised two-period cross-over trial (*Matzneller et al*) evaluating the pharmacokinetic profile of a new flurbiprofen 8.75 mg compressed lozenge as compared to the marketed flurbiprofen 8.75 mg lozenge (Matzneller et al., 2012). The event was reported as “haematoma” but no further information on the site or size of the haematoma was specified, although it was reported by the authors as “non-serious, not related to treatment and of mild intensity”. As this study was a cross-over trial with all 12 patients taking the active and comparator drug and with no further information on the onset of the event, it is not possible to compare the frequency of the haemorrhagic events with a comparator. However, as both active and comparator drugs were flurbiprofen 8.75 mg lozenges this study does not provide a true comparator for which an assessment of differences in risk of haemorrhagic events can be made.

The remaining two records (NCT01048866 (ClinicalTrials.gov. National Library of Medicine (US), 2010a) and NCT01049334 (ClinicalTrials.gov. National Library of Medicine (US), 2010b)) reporting haemorrhagic events were both randomized double-blind placebo-controlled trials comparing the safety and efficacy of flurbiprofen 8.75 mg lozenge to a placebo lozenge for the treatment of painful pharyngitis. For NCT01048866, risk of haemorrhagic events was 1.98% with flurbiprofen 8.75 mg lozenge vs 2.06% in the placebo arm. Risks were calculated as the number of haemorrhagic events/number of patients at risk as an individual patient may have experienced more than one haemorrhagic event. In the third trial (NCT01049334), corresponding risk of haemorrhagic events was 1.96% and 0.98%, respectively. Comparison of flurbiprofen 8.75 mg lozenge to placebo produced contrasting risk ratios of 0.96 (95% CI 0.07, 13.25) in NCT01048866 and 2.00 (95% CI 0.10, 118.0) in NCT01049334, despite identical methods used in both studies. These effect measures were not provided by the authors but calculated for both studies. However, CIs were wide and overlapping and thus results were not statistically significant. Sample sizes and event counts were small, hence likely susceptible to a high degree of random error and observed results may be due to chance.

When comparing across treatment groups, there was some consistency in the nature of the events in the two trials, NCT01048866 and NCT01049334. Haemorrhagic events in NCT01048866 were reported as “haematochezia” and “epistaxis” in the flurbiprofen arm and “mouth haemorrhage” and “epistaxis” in the placebo arm. In NCT01049334 haemorrhagic events were “epistaxis” in both the flurbiprofen and placebo treatment arms. All events in both trials were classified as non-serious as reported by the authors and the causality was not documented. Although GI bleeding has been reported with all NSAIDs, it is not usually observed with short term limited use products such as flurbiprofen lozenges and has not been listed as an adverse event experienced with flurbiprofen at OTC doses for short term use (Reckitt Benckiser Healthcare (UK) Ltd, 2016). It is possible that patients with a history of GI disease may be at higher risk, however these studies excluded patients with a history of upper gastric ulcer within the past 60 days, or patients experiencing significant upper GI complaints. In addition, the GI haemorrhagic event of “haematochezia” in the flurbiprofen arm in NCT01048866 is more indicative of bleeding from the lower GI tract (with multiple potential aetiologies) but may in some circumstances result from significant upper GI bleeding. However, the event was classified as non-serious as reported by the authors indicating it is more likely to be the former. Epistaxis, reported in patients taking flurbiprofen 8.75 mg in both trials, has not been documented as an adverse event relating to flurbiprofen OTC use (Reckitt Benckiser Healthcare (UK) Ltd, 2016).

Despite low counts of haemorrhagic events across all trials, small study sample sizes yielded a common incidence (i.e., ≥1.0%) of haemorrhagic events with flurbiprofen 8.75 mg. However, all haemorrhagic events occurring with flurbiprofen 8.75 mg were reported by the authors as non-serious and none were confirmed to be causally related; one was reported as not-related and for the remaining causality was not provided.

Additional secondary objectives of this systematic review included describing the frequency of haemorrhagic events as a result of potential DDIs and describing the nature of the DDI. Exclusion criteria for the study by *Matzneller et al* included use of any medicinal product within 14 days prior to study start, treatment with any known enzyme-inhibiting or -inducing agents within 4 weeks prior to study start, participation in an ongoing trial or previous clinical trial within 3 months prior to the study start. Thus, from the exclusion criteria it is likely that the haemorrhagic event occurred in the absence of a DDI, however individual medication history for the patient experiencing the haemorrhagic event is not known.

Exclusion criteria for NCT01048866 and NCT01049334 were identical and included patients taking regular medication (≥three times in the previous week) and a history of chronic analgesic use (≥three times per week over the prior 4 weeks). Patients on low dose aspirin therapy and women taking contraception were allowed in both studies. The product information for flurbiprofen advises avoidance with concomitant medications which may increase the risk of bleeding, such as other NSAIDs and acetylsalicylic acid (including low dose) and caution with use of oral corticosteroids, anticoagulants, selective serotonin-reuptake inhibitors. Nevertheless, it is noted that undesirable effects may be minimized by using the lowest effective dose of flurbiprofen for the shortest duration necessary to control symptoms (Reckitt Benckiser Healthcare (UK) Ltd, 2016). Both trials included flurbiprofen 8.75 mg lozenge as required for a duration of 7 days. Further information on the exact use of concomitant medication in the individuals enrolled in both studies is not available, thus it is not possible to further comment on these events in the context of DDIs.

Where additional haemorrhagic events were identified with low doses of flurbiprofen, these were observed in patients undergoing oral surgery (e.g., tonsillectomy, palatal graft harvesting surgery, mandibular extraction) (Türk et al., 2018; Isler et al., 2018; Dionne et al., 2004). Bleeding events were described as post-operative haemorrhage or delayed bleeding. With such indications and haemorrhagic events, there is potential for confounding by indication; for all three studies the authors did not report suspected causality with flurbiprofen. Comparator groups across the three studies included oral NSAID (ibuprofen), oral flurbiprofen at higher doses or placebo. From the results of these studies, it is not possible to conclude a difference in risk of bleeding with flurbiprofen low dose vs comparator.

### Strengths

To date, although the risk with higher doses of flurbiprofen is known, to our knowledge no systematic review on the risk of haemorrhagic events with flurbiprofen 8.75 mg lozenge/oromucosal spray has been published. In this systematic review, a thorough approach was used with a broad search strategy across multiple databases. Search terms in this review were comprehensive including (where possible) all synonyms for flurbiprofen and the formulation of interest. Published and unpublished (e.g., ClinicalTrials.gov) data sources were examined. All records were reviewed by two reviewers independently and where necessary adjudicated by a third reviewer, thus minimising the potential for reporting bias.

### Limitations

As with all systematic reviews, there are a number of limitations; some relate to the review process itself and others relate to the studies included in the review.

The aim of this systematic review was to identify all eligible studies. Thus, it is unlikely that the review captured a biased sample of studies as data was identified from both published and unpublished studies. However, due to the paucity of data, no additional analyses were undertaken to assess this bias. Potential biases in the review process include only reviewing English language studies. The included studies were based in Europe and the United States, thus may not be generalisable to other populations but there is little reason to believe that this drug would have different effects in other geographical areas. Furthermore, conference abstracts were not included; this represents a potential publication bias. It is also acknowledged that smaller uneventful studies, both in terms of efficacy and safety, are not always published. In addition, only a limited number of studies have been conducted and reported for flurbiprofen at lower doses, specifically the 8.75 mg dose. Haemorrhagic events (and corresponding details related to the event) included in this systematic review were those available in the public domain (i.e., reported in publications). In terms of limitations arising from the included studies and other studies reporting 8.75 mg flurbiprofen use, the most significant limitation was that studies were not specifically designed and thus powered to investigate haemorrhagic events. Potential for under-reporting of haemorrhagic events is possible in studies that were not designed to investigate this outcome, thus results of this systematic review may not be an accurate reflection of the overall risk.

There is also potential for reporter and/or observer bias leading to differential misclassification of the outcome in open-label trials and observational studies; for these studies, knowledge of use of the lower dose of flurbiprofen may have led to expectation bias and subsequently affected patient reporting and/or investigator data collection.

Due to limited information available at the case level, further exploration of the risk of haemorrhagic events in the context of potential DDIs was not possible. Furthermore, although exclusion criteria for the studies included specific categories of past medical history (e.g., upper gastrointestinal ulcer, history of hepatic disease), individual bleeding risk factors at the case level were not known. Plasma drug levels were also not available in the context of haemorrhagic events. Thus, there is insufficient information to assess whether a causal relationship exists between flurbiprofen 8.75 mg and the haemorrhagic events reported in the included studies.

Only three studies were considered eligible for the systematic review and in each study sample size and event counts were small. For some studies (not included), only adverse events were specified with no further details on the nature of the event. However, if a haemorrhagic event was identified in the study itself, given its potential clinical significance it may be inferred that the absence of reporting of a haemorrhage event in the paper implies that the event did not occur. Where a haemorrhagic event was reported in the included eligible study, limited clinical information on the event was provided, in addition to a lack of formal statistical comparisons across treatment groups.

Finally, in terms of quality of evidence, risk of bias was difficult to assess for all three studies included as the studies and the corresponding details provided were tailored towards assessing either the pharmacokinetic profile or efficacy. All three studies were randomized controlled studies; two of which were double-blind thus reducing the potential for both selection bias and information bias respectively. However, based on all information available and following the systematic Cochrane risk of bias tool assessment (RoB2), all three studies were concluded as having “some concerns” with regards to risk of bias. This result was predominantly driven by a lack of information (accessible in the public domain) on a pre-specified analysis plan for safety, in particular haemorrhagic outcomes, as these were not the primary outcomes of interest in the studies reviewed. Furthermore, the certainty of evidence for the safety outcome of haemorrhagic events was assessed as “very low” using the GRADE framework. Although the study design of randomised trials produced an initial high quality of evidence, the domains of indirectness and imprecision decreased the level of certainty.

To identify haemorrhagic events not captured in published studies identified from the initial search, a review of EudraVigilance and the EMA website was performed. Examination of EudraVigilance was limited by a lack of granularity on flurbiprofen dose/formulation and no cases were identified from the EMA website. A further exhaustive search of grey literature was not performed due to resource limitations. However, the limited grey literature search performed was per protocol, as it was considered unlikely that further review of grey literature would identify sufficient new information on this subject to impact the results of the systematic review.

## Conclusion

In conclusion, this systematic review found limited evidence on the risk of haemorrhagic events with flurbiprofen 8.75 mg. Of the records retrieved from the electronic database search specifically examining 8.75 mg flurbiprofen, only three studies reported haemorrhagic events. Counts of haemorrhagic events were low in patients receiving flurbiprofen across all three studies; all were reported as non-serious and comparison between flurbiprofen and placebo treatment arms was non-significant.

However, scarcity of studies and low certainty of evidence for the outcome of haemorrhagic events limits the conclusions of this systematic review. Further well-designed research is recommended to investigate the risk of haemorrhage further. This may include real-world observational studies such as post-authorisation safety studies (PASS) designed to specifically investigate haemorrhagic outcomes with flurbiprofen 8.75 mg.

## Data Availability

The original contributions presented in the study are included in the article/Supplementary Material, further inquiries can be directed to the corresponding author.

## References

[B1] Balshem H. Helfand M. Schünemann H. J. Oxman A. D. Kunz R. Brozek J. (2011). Grade Guidelines: 3. Rating the Quality of Evidence. J. Clin. Epidemiol. 64, 401–406. 10.1016/j.jclinepi.2010.07.015 21208779

[B2] Clinicaltrials.Gov. National Library Of Medicine (Us). 2010a. Nct01048866. A Repeat-Dose, Multi-Centre, Randomised, Double-Blind, Placebo-Controlled, Study to Determine the Safety and Efficacy of Flurbiprofen 8.75mg Lozenge Compared to its Vehicle Control Lozenge in Patients with Painful Pharyngitis Available at: https://Clinicaltrials.Gov/Ct2/Show/Nct01048866 (Accessed 30 April 2020).

[B3] Clinicaltrials.Gov. National Library Of Medicine (Us) (2010b). Nct01049334. A Randomized, Double-Blind, Placebo-Controlled Multiple-Dose Study to Determine the Efficacy, Onset, and Duration of Action of Flurbiprofen 8.75 Mg Lozenge Compared to its Vehicle Control Lozenge in Patients with Painful Pharyngitis. Available at: https://Clinicaltrials.Gov/Ct2/Show/Nct01049334 (Accessed April 30, 2020).

[B4] Dionne R. A. Haynes D. Brahim J. S. Rowan J. S. Guivarc'h P.-H. (2004). Analgesic Effect of Sustained-Release Flurbiprofen Administered at the Site of Tissue Injury in the Oral Surgery Model. J. Clin. Pharmacol. 44, 1418–1424. 10.1177/0091270004265703 15545314

[B5] Gulliford M. C. Dregan A. Moore M. V. Ashworth M. Staa T. V. Mccann G. (2014). Continued High Rates of Antibiotic Prescribing to Adults with Respiratory Tract Infection: Survey of 568 Uk General Practices. Bmj Open 4, E006245. 10.1136/bmjopen-2014-006245 PMC4212213 25348424

[B6] Higgins J. Thomas J. (2019). Cochrane Handbook for Systematic Review of Interventions. Version 6. Chicester: The Cochrane Collaboration.

[B7] Isler S. C. Eraydin N. Akkale H. Ozdemir B. (2018). Oral Flurbiprofen Spray for Mucosal Graft Harvesting at the Palatal Area: A Randomized Placebo-Controlled Study. J. Periodontol. 89, 1174–1183. 10.1002/jper.17-0381 30007054

[B8] Liberati A. Altman D. G. Tetzlaff J. Mulrow C. Gotzsche P. C. Ioannidis J. P. A. (2009). The Prisma Statement for Reporting Systematic Reviews and Meta-Analyses of Studies that Evaluate Healthcare Interventions: Explanation and Elaboration. Bmj 339, B2700. 10.1136/bmj.b2700 19622552 PMC2714672

[B9] Matzneller P. Burian A. Martin W. Annoni O. Lauro V. Tacchi R. (2012). A Randomised, Two-Period, Cross-Over, Open-Label Study to Evaluate the Pharmacokinetic Profiles of Single Doses of Two Different Flurbiprofen 8.75-Mg Lozenges in Healthy Volunteers. Pharmacology 89, 188–191. 10.1159/000336767 22433300

[B10] Moore N. Pollack C. Butkerait P. (2015). Adverse Drug Reactions and Drug-Drug Interactions with Over-the-counter Nsaids. Ther. Clin. Risk Manag. 11, 1061–1075. 10.2147/TCRM.S79135 26203254 PMC4508078

[B11] Murad M. H. Sultan S. Haffar S. Bazerbachi F. (2018). Methodological Quality and Synthesis of Case Series and Case Reports. Bmj Ebm 23, 60–63. 10.1136/bmjebm-2017-110853 PMC6234235 29420178

[B12] National Institute For Health And Care Excellence (2018). Nice Guideline [Ng84]. Sore Throat (Acute): Antimicrobial Prescribing. Available: https://Www.Nice.Org.Uk/Guidance/Ng84 (Accessed March 10, 2020).

[B13] Page M. J. Mckenzie J. E. Bossuyt P. M. Boutron I. Hoffmann T. C. Mulrow C. D. (2021). The Prisma 2020 Statement: An Updated Guideline for Reporting Systematic Reviews. Bmj 372, N71. 10.1136/bmj.n71 33782057 PMC8005924

[B14] Rao P. Knaus E. E. (2008). Evolution of Nonsteroidal Anti-inflammatory Drugs (Nsaids): Cyclooxygenase (Cox) Inhibition and beyond. J. Pharm. Pharm. Sci. 11, 81s–110s. 10.18433/j3t886 19203472

[B15] Reckitt Benckiser Healthcare (Uk) Ltd 2016. Summary of Product Characteristics. Flurbiprofen 8.75mg Strefen Honey And Lemon.

[B16] Reckitt Benckiser Healthcare (Uk) Ltd (2018). Summary of Product Characteristics. Strefen Direct 8. 75mg/Dose Oromucosal Spray, Solution.

[B17] Ricciotti E. Fitzgerald G. A. (2011). Prostaglandins and Inflammation. Atvb 31, 986–1000. 10.1161/atvbaha.110.207449 PMC3081099 21508345

[B18] Sterne J. A. C. Savović J. Page M. J. Elbers R. G. Blencowe N. S. Boutron I. (2019). Rob 2: A Revised Tool for Assessing Risk of Bias in Randomised Trials. Bmj 366, L4898. 10.1136/bmj.l4898 31462531

[B19] Sterne J. A. Hernán M. A. Reeves B. C. Savović J. Berkman N. D. Viswanathan M. (2016). Robins-I: A Tool for Assessing Risk of Bias in Non-randomised Studies of Interventions. Bmj 355, I4919. 10.1136/bmj.i4919 27733354 PMC5062054

[B20] Türk B. Akpınar M. Erol Z. N. Kaya K. S. Ünsal Ö. Coşkun B. U. (2018). The Effect of Flurbiprofen Oral Spray and Ibuprofen vs Ibuprofen Alone on Postoperative Tonsillectomy Pain: An Open, Randomised, Controlled Trial. Clin. Otolaryngol. 43, 835–840. 10.1111/coa.13058 29288561

