# Supplementary material for: A Systematic Review of Flurbiprofen 8.75 mg Dose and Risk of Haemorrhagic Events
Source: Front Pharmacol. 2021 Aug 4;12:726141. doi: 10.3389/fphar.2021.726141 (PMC8371335; doi:10.3389/fphar.2021.726141)
Supplement: Supplementary file 1 [file DataSheet1.PDF]

Supplementary Material

Search Strategy

Pubmed

Filters applied - English language, Humans

Search concept(s) -

(Flurbiprofen OR Strepfen OR Strepsils OR Strefen OR Benactiv Gola OR Strepflam OR Graneodin-F OR СТРЕПФЕН OR Стрепсилс® Интенсив OR Strepsilsmaxpro OR Streflam OR Verofen OR Buccostad OR Sorenex OR Geifen OR Camilfen OR Sereno OR Ultravox Maxe OR Strefzap OR Benactivdol Gola OR Froben)

AND

(spray OR oral spray OR oromucosal spray OR mucosal spray OR mucosal OR oromucosal OR lozenge* OR buccal OR sublingual OR pastille* OR topical OR granule* OR oral solution OR mouthwash OR 8.75*)

Embase

Filters applied – English language, Humans, Articles

Search concept(s) -

(Flurbiprofen OR Strepfen OR Strepsils OR Strefen OR “Benactiv Gola” OR Strepflam OR Graneodin-F OR СТРЕПФЕН OR “Стрепсилс® Интенсив” OR Strepsilsmaxpro OR Streflam OR Verofen OR Buccostad OR Sorenex OR Geifen OR Camilfen OR Sereno OR “Ultravox Maxe” OR Strefzap OR “Benactivdol Gola” OR Froben)

AND

(spray OR “oral spray” OR “oromucosal spray” OR “mucosal spray” OR mucosal OR oromucosal OR lozenge* OR buccal OR sublingual OR pastille* OR topical OR granule* OR “oral solution” OR mouthwash OR 8.75*)

Web of Science

Filters applied - English language, Articles

Search concept(s) -

(Flurbiprofen OR Strepfen OR Strepsils OR Strefen OR Benactiv Gola OR Strepflam OR Graneodin-F OR СТРЕПФЕН OR Стрепсилс® Интенсив OR Strepsilsmaxpro OR Streflam OR Verofen OR Buccostad OR Sorenex OR Geifen OR Camilfen OR Sereno OR Ultravox Maxe OR Strefzap OR Benactivdol Gola OR Froben)

AND

(spray OR oral spray OR oromucosal spray OR mucosal spray OR mucosal OR oromucosal OR lozenge* OR buccal OR sublingual OR pastille* OR topical OR granule* OR oral solution OR mouthwash OR 8.75*)

<u>Cochrane</u>

Filters applied – Trials

Search concept(s) -

(Flurbiprofen OR Strepfen OR Strepsils OR Strefen OR Benactiv Gola OR Strepflam OR Graneodin-F OR СТРЕПФЕН OR Стрепсилс® Интенсив OR Strepsilsmaxpro OR Streflam OR Verofen OR Buccostad OR Sorenex OR Geifen OR Camilfen OR Sereno OR Ultravox Maxe OR Strefzap OR Benactivdol Gola OR Froben)

AND

(spray OR oral spray OR oromucosal spray OR mucosal spray OR mucosal OR oromucosal OR lozenge* OR buccal OR sublingual OR pastille* OR topical OR granule* OR oral solution OR mouthwash OR 8.75*)

<u>Clinical trials.gov</u>

Filters applied- none

Search concept(s) -

Flurbiprofen

<u>EU clinical trials.gov</u>

Filters applied – none

Search concept(s) -

(Flurbiprofen OR Strepfen OR Strepsils OR Strefen OR Benactiv Gola OR Strepflam OR Graneodin-F OR СТРЕПФЕН OR Стрепсилс® Интенсив OR Strepsilsmaxpro OR Streflam OR Verofen OR Buccostad OR Sorenex OR Geifen OR Camilfen OR Sereno OR Ultravox Maxe OR Strefzap OR Benactivdol Gola OR Froben)

AND

(spray OR oral spray OR oromucosal spray OR mucosal spray OR mucosal OR oromucosal OR lozenge* OR buccal OR sublingual OR pastille* OR topical OR granule* OR oral solution OR mouthwash OR 8.75*)

<u>PubMed case reports</u>

Filter applied- English language, Humans, Case reports

Search concept(s) -

(Flurbiprofen OR Strepfen OR Strepsils OR Strefen OR Benactiv Gola OR Strepflam OR Graneodin-F OR СТРЕПФЕН OR Стрепсилс® Интенсив OR Strepsilsmaxpro OR Streflam OR Verofen OR Buccostad OR Sorenex OR Geifen OR Camilfen OR Sereno OR Ultravox Maxe OR Strefzap OR Benactivdol Gola OR Froben)

Data items for extraction

Data	Result
**Information on paper**	
Study ID<br>Name of first author<br>Year of publication<br>Title of paper<br>Link to local pdf copy or hyperlink to online copy	Allocated by reviewer
**Study design**	
Type of study (e.g. RCT, cohort, case control)<br>Country<br>Setting of study<br>Follow-up duration (if applicable)	Primary, secondary care
**Population studied**	
Specific age criteria to study population (if any)<br>Medical indication for treatment	
**Exposure of interest**	
Flurbiprofen (dose confirmation, formulation)	
**Comparator**	
Placebo or active comparator<br>If active - class of medicinal product, name, dose, formulation, indication for prescribing, category of sales (GSL, PO, POM), alone or in combination with other medicinal product	
**Baseline results**	
Total sample size<br>Number of participants taking flurbiprofen<br>Number of participants taking comparator<br>Age of participants<br>Gender<br>Relevant co-morbidities	
**Method of statistical analysis**	
Crude/adjusted measures<br>Modelling<br>Adjustment for confounding	
**Outcomes**	
Frequency of events and in which group of patients (exposure or comparator if comparator applicable)<br>Details of events – site, severity, clinical sequelae<br>Details of whether event occurred with exposure or comparator alone or whether in combination with other medicinal products (i.e. DDI's)<br>If occurred in combination with other medicinal product - details of concomitant medications (class, dose, formulation, indication)	
**Measure of frequency/effect**	
Risk of event (in either group)<br>Rate/odds of event (if provided)<br>Measure of effect (crude and/or adjusted) – risk ratio, rate ratio, odds ratio, hazard ratio (depending on type of study and what has been reported)<br>Confidence interval (if provided)	

p-value (if provided)	
**Author reported causality[a]**	

[a] Author reported causality will be extracted if provided; otherwise individual causality assessments will not be performed as part of this systematic review

Results of eligible studies from electronic database search

1. Matzneller et al

A randomised, two-period, cross-over, open-label study evaluated the pharmacokinetic profiles of two different oromucosal flurbiprofen 8.75mg lozenges in healthy volunteers aged between 18-55 years.

Twelve healthy volunteers were enrolled to the study and randomised to receive the test drug, the new flurbiprofen 8.75mg compressed lozenges (Alfa Wasserman S.p.A), or the marketed flurbiprofen 8.75mg lozenges (Benactiv Gola®, Reckitt Benckiser Healthcare). Each volunteer was administered two lozenges (to be sucked and slowly dissolved in the mouth) at point of randomisation and blood samples were collected at pre-defined intervals for 24 hours after the dose. A washout period of at least seven days separated the two study periods (i.e., before the subject was administered the alternative lozenge).

Results from this study report one haemorrhagic event, specified as 'haematoma'; risk 8.3% (n=1)[1]. The onset of the event in relation to the study drugs was not reported, thus it is not known after which lozenge the event occurred and after how long. The authors described as the event as 'not serious, not related to treatment or of mild intensity'. No further information on the haematoma was provided.

2. NCT01048866

This multi-centre randomised double-blind placebo-controlled trial compared the safety and efficacy of flurbiprofen 8.75mg lozenge to placebo lozenge in patients > 18 years old with painful pharyngitis.

In total, 198 patients were enrolled across four different sites; 101 patients were allocated to the flurbiprofen treatment arm and 97 to the placebo arm. Participants were instructed to suck one lozenge (flurbiprofen or placebo) every 3-6 hours, up to a total of five lozenges in 24 hours for the seven days of the study. Patients were also allowed a dose of rescue medicine (acetaminophen 650mg), as needed. A diary was provided to each patient to record their safety and efficacy assessment immediately before and after each lozenge was taken, as required, over the seven-day treatment period.

Adverse events were assessed from the initial dose on day one up to day seven. The events were collected by systematic assessment and an individual patient may have experienced more than one adverse event. In the flurbiprofen lozenge arm there were two cases of bleeding events; these were reported[2] as 'haematochezia' (n=1, 0.99%) and 'epistaxis' (n=1, 0.99%). In the placebo lozenge arm, there were also two bleeding events reported as 'mouth haemorrhage' (n=1, 1.03%) and 'epistaxis' (n=1, 1.03%). The events were classified as non-serious. No further information was provided on these specific bleeding events in the public domain.

3. NCT01049334

[1] Risk not reported in paper but calculated from numerator and denominator information.
[2] Term from vocabulary, MedDRA (v14.0)

This single-centre randomised double-blind placebo-controlled trial compared the analgesic efficacy of flurbiprofen 8.75mg lozenge to placebo lozenge and the safety of flurbiprofen throughout the course of treatment of sore throat due to acute pharyngitis.

In total, 204 patients were enrolled; 102 patients were allocated to the flurbiprofen group and 102 to the placebo group. Participants were advised to suck one lozenge (flurbiprofen or placebo) every 3-6 hours, up to a total of five lozenges a day, as needed for pain for seven days.

Adverse events were assessed from the initial dose on day one up to day seven. The events were collected by systematic assessment and an individual patient may have experienced more than one adverse event. In the flurbiprofen lozenge arm there were two cases of bleeding events; these were reported[3] as 'epistaxis' (n=2, 1.96%). In the placebo lozenge arm, there was also one case of 'epistaxis' (n=1, 0.98%). All adverse events were classified as non-serious. No further bleeding events were reported in either treatment arm and no further information on the nature of the adverse event was provided in the publication[4].

[3] Term from vocabulary, MedDRA (v14.0)
[4]Publications automatically indexed to this trial on ClinicalTrials.gov were reviewed but no further information relating to the nature of these adverse events was provided.

GRADE Evidence Profile Flurbiprofen 8.75mg compared to placebo

Certainty assessment							№ of patients		Effect		Certainty	Importance
№ of studies	Study design	Risk of bias	Inconsistency	Indirectness	Imprecision	Other considerations	Flurbiprofen 8.75mg	Placebo	Relative (95% CI)	Absolute (95% CI)		
Haemorrhagic events (follow up: range 1 days to 7 days)												
3	randomised trials	not serious	not serious	very serious [a]	serious [b]	publication bias strongly suspected [c]	5/215 (2.3%)	3/199 (1.5%)	not pooled	see comment	⨁◯◯◯ VERY LOW	IMPORTANT

CI: Confidence interval

a. Studies included were not specifically designed and powered to investigate haemorrhagic events.

b. Effect estimates were only available for two studies with few events

c. Conference abstracts were not included and it is also acknowledged that smaller uneventful studies, both in terms of efficacy and safety, are not always published.
